# Attitudes Toward Deprescribing Among Older Adults Seeking Care in the Emergency Department

**DOI:** 10.1016/j.acepjo.2026.100467

**Published:** 2026-07-28

**Authors:** Emma Lopes, Grace Burud, Smeet Bhimani, Parag Goyal, Joshua Niznik, Kaitlin Donovan, Andrew Dodson, Greta Anton, Jan Busby-Whitehead, Michelle L. Meyer, Martin F. Casey

**Affiliations:** 1 University of North Carolina School of Medicine, Chapel Hill, North Carolina, USA; 2 Department of Emergency Medicine, University of North Carolina School of Medicine, Chapel Hill, North Carolina, USA; 3 Department of Medicine, Weill Cornell Medicine, New York, New York, USA; 4 Division of Geriatric Medicine and Center for Aging and Health, University of North Carolina at Chapel Hill, School of Medicine, Chapel Hill, North Carolina, USA; 5 Division of Pharmaceutical Outcomes and Policy, University of North Carolina at Chapel Hill, Eshelman School of Pharmacy, Chapel Hill, North Carolina, USA; 6 Center for Health Equity Research and Promotion, Veterans Affairs (VA) Pittsburgh Healthcare System, Pittsburgh, Pennsylvania, USA; 7 Eshelman School of Pharmacy, University of North Carolina at Chapel Hill, Chapel Hill, North Carolina, USA; 8 Department of Pharmacy, University of North Carolina Medical Center, Chapel Hill, North Carolina, USA

**Keywords:** deprescribing, geriatric patients, adverse drug events

## Abstract

**Objectives:**

Deprescribing potentially unnecessary or harmful medications may help reduce avoidable emergency department (ED) visits for adverse drug events, which are prevalent among older adults. However, deprescribing in the ED remains rare, in part due to limited evidence on how to implement such practices and an incomplete understanding of patient attitudes in this setting. This study aimed to assess attitudes toward deprescribing among older adults presenting to the ED and explore willingness to accept deprescribing recommendations from emergency clinicians.

**Methods:**

We conducted an observational study of adults aged ≥65 years at a single academic ED from June to October 2024. Participants completed the Revised Patients’ Attitudes Towards Deprescribing (rPATD) questionnaire, which evaluates general willingness to deprescribe and perceived barriers across four domains: medication burden, perceived appropriateness, concerns about stopping, and involvement in decision making. An exploratory question assessed willingness to deprescribe if recommended by ED staff during an ED visit. Descriptive statistics and 95% CIs were calculated.

**Results:**

We enrolled 116 older adults presenting to the ED who completed the rPATD questionnaire. Median patient age was 75 years (IQR 69-80), 72% had multimorbidity, and 56% reported polypharmacy (≥5 medications). Most participants expressed willingness to deprescribe if recommended by their prescriber (91%; 95% CI: 84-95). In contrast, 53% (95% CI: 43-62) expressed reluctance to stop long-standing medications. Most participants reported satisfaction with current medications (87%; 95% CI: 80-92) and a low perceived medication burden, although 28% (95% CI: 21-37) acknowledged possible side effects. Additionally, 87% (95% CI: 80-92%) expressed a desire to be involved in medication-related decisions. Lastly, in an ED-specific exploratory item, 79% of participants (95% CI: 71-86) expressed willingness to deprescribe when recommended by ED staff.

**Conclusion:**

Older adults presenting to the ED are highly receptive to deprescribing, including when recommended by ED staff. These findings support the feasibility of ED-based deprescribing interventions, particularly those that incorporate shared decision making and coordination with outpatient care.

**Bottom Line**

Older adults frequently experience medication-related harm, yet little is known about whether they would accept deprescribing recommendations during an emergency department (ED) visit. In this survey study of 116 adults aged 65 years and older presenting to a single academic ED, a validated questionnaire found that most participants were willing to stop ≥1 medications if recommended by their usual prescriber (91%) and were also receptive to recommendations from an emergency clinician (79%). These findings suggest that patient acceptance may not be a major barrier to emergency department-based deprescribing and support the ED as an important setting to initiate deprescribing conversations and reduce medication-related harm.

## 1 Introduction

### 1.1 Background

Adverse drug events (ADEs) continue to place a significant burden on the US health care system, resulting in approximately 450,000 to 550,000 emergency department (ED) visits annually among adults aged ≥65 years.^1^^,^^2^ Notably, an estimated 70% of these ADE-related ED visits are considered preventable.^3^ One promising strategy to reduce preventable ADEs is deprescribing—the process of intentionally discontinuing medications that may be harmful or no longer beneficial.^3^^,^^4^

### 1.2 Importance

Despite its potential, deprescribing in the ED remains uncommon even during encounters for ADEs, which represent missed opportunities to address problematic medication use.^5^ ED staff often express reluctance to initiate deprescribing, citing barriers, such as concerns about encroaching on other providers when adjusting medications and the challenge of reviewing complete medication lists.^6^ Although ED staff report significant barriers, little is known about whether older adults themselves would accept deprescribing recommendations during ED encounters.

### 1.3 Goals of This Investigation

To address this gap, we assessed older adults’ attitudes toward deprescribing during ED visits. We administered the Revised Patients' Attitudes Towards Deprescribing (rPATD) survey, a validated instrument used across diverse populations in the primary care setting.^7^ Unlike the original Patients' Attitudes Towards Deprescribing, the rPATD is a more concise instrument that assesses willingness to deprescribe by identifying barriers across four domains: medication burden, perceived appropriateness, concerns about stopping, and involvement in medication decisions. Critically, we augmented this tool with an ED-specific exploratory item to gauge patient willingness to accept recommendations initiated specifically by ED staff. This approach allows us to assess whether older adults are receptive to deprescribing recommendations initiated in the ED, a setting in which patient attitudes are poorly understood.

## 2 Methods

### 2.1 Study Design and Participants

We conducted an observational study at the University of North Carolina Medical Center ED, a level I academic trauma center, that serves both a local college-town population and a broad statewide catchment area. The study was conducted from June to October 2024. This study was reviewed and deemed exempt by the University of North Carolina Institutional Review Board (IRB #24-1128), with a waiver of written consent granted; verbal consent was obtained from all participants.

We recruited a convenience sample of eligible patients presenting between 9:00 AM and 9:00 PM, Monday through Friday, based on study personnel availability. Initial screening to identify eligible patients was conducted using the Epic electronic health record system. Patients aged ≥65 years presenting to the ED were eligible for inclusion. We excluded non-English-speaking patients, those with cognitive impairment or with behavioral concerns, and patients whose clinical status (eg, resuscitation and urgent procedures) made participation infeasible. Cognitive impairment was defined as a documented diagnosis of Alzheimer disease or related dementia in the electronic medical record, based on diagnostic codes consistent with the Chronic Conditions Warehouse definition of dementia.^8^ Behavior concerns were determined at the discretion of the treating attending ED physician and were, therefore, subjective. Attendings were instructed to exclude patients with intoxication or aggressive behavior. Once screened, eligible patients were approached by research personnel in the ED during ongoing clinical workups prior to disposition. The rPATD survey, which is the focus of this paper, was coadministered with a partner survey examining patient communication preferences for aspirin management during bleeding encounters.^9^ The rPATD survey broadly assesses deprescribing attitudes, whereas the partner survey explored patient preferences in the specific context of a manifested adverse drug event (namely aspirin-related bleeding). For each encounter, the rPATD survey was administered first. Because the partner survey addressed active bleeding scenarios and was administered second, we excluded patients presenting with medication-related hemorrhage to avoid interfering with standard clinical care. Verbal consent was obtained from all participants. Patients who were invited and consented agreed to participate in both surveys. The questionnaire for rPATD survey is provided in Figure S1.

Surveys were administered via a printed questionnaire, completed independently by participants or with assistance from research personnel in reading and writing. Instructions for completing the survey were provided verbally and in writing on the printed questionnaire by research personnel. All patients were informed prior to administration that participation in the survey was voluntary and would not affect their care in any way. Both surveys required approximately 10 to 20 minutes to complete. Data were collected and stored using REDCap, a secure, web-based software platform that complies with the Heath Insurance Portability and Accountability Act regulations and electronic data collection.

### 2.2 Data Collection

Before administering the survey, demographic information from the participants was collected, including age, gender, race, ethnicity, level of education, and residence. Additionally, we collected self-reported medical history on multimorbidity (defined as ≥2 chronic conditions)^10^ and polypharmacy (defined as no polypharmacy if ≤4 prescriptions, polypharmacy if 5-9, and hyper polypharmacy if ≥10).^11^

This study used versions of the validated rPATD questionnaire to assess the attitudes, beliefs, and experiences related to deprescribing within the targeted populations. Developed and validated in Australia in 2016 for older adults, the rPATD has since then been utilized internationally, including in the United States.^12^, ^13^, ^14^, ^15^, ^16^ It has been previously implemented in outpatient and inpatient care settings, as well as among community-dwelling older adults. The rPATD has demonstrated face, content, criterion, construct, and internal validity among older adults.^17^ Permission to use the questionnaire in this study was obtained from the original developers.

The rPATD survey comprises 24 statements (Fig S1), including two global questions about overall satisfaction with medication use and willingness to accept deprescribing recommendations. The remaining 22 questions addressed four subdomains that may present barriers to deprescribing including: (1) perceived medication burden (*Burden factor*), (2) attitudes toward the appropriateness of prescribed medications (*Appropriateness factor*), (3) concerns about stopping medications (*Concerns about stopping factor*), and (4) participants’ knowledge about their medications and their preference to be involved in the medication decision-making process (*Involvement factor*).^7^ All 24 questions in the rPATD are scored on a 5-point Likert scale. (1 = strongly disagree, 2 = disagree, 3 = unsure, 4 = agree, and 5 = strongly agree). It is essential to note that all questions in the rPATD pertain to a potential conversation with a subject’s prescriber, rather than ED staff. To further explore our subjects’ willingness to accept deprescribing recommendations from ED staff, we added an exploratory question: “If my emergency provider said one or more of my medicines were causing problems, I would be willing to stop them.” This exploratory question is not part of the validated rPATD instrument, was analyzed separately from the rPATD responses, and should not be interpreted as equivalent to its standardized constructs. To preserve the validity of the original rPATD and minimize any potential influence on responses, the exploratory question was placed at the end of the survey.

### 2.3 Data Analysis

Descriptive statistics were applied to characterize study participants and describe their attitudes toward deprescribing. Frequencies were reported along with Wald (binomial-normal approximation) 95% CIs for the proportion of patients who reported “strongly agree” or “strongly disagree” for the primary and secondary outcomes. In our primary statistical test, we sought to describe whether a majority of patients demonstrated a willingness to deprescribe by answering either “strongly agree” or “agree” to the global rPATD question: “If my doctor said it was possible I would be willing to stop one or more of my regular medicines.” Unfortunately, there are no widely accepted reporting standards when describing proportions or prevalence. Study authors defined a "majority" as a proportion where the lower bound of the 95% CI >50%, and a "strong majority" as a proportion where the lower bound of the 95% CI >67%. These thresholds were chosen pragmatically to provide interpretive clarity and are not based on established reporting guidelines; they should be considered exploratory. Additionally, we conducted exploratory bivariate analyses examining associations between participants’ willingness to deprescribe and individual demographic and clinical characteristics using chi-squared tests. All statistical analyses were conducted in StataNow 18 BE (StataCorp LLC).

## 3 Results

### 3.1 Participant Characteristics

A total of 585 individuals were screened for participation. Among those screened, 179 (31%) were excluded for severity-related exclusions (eg, ongoing resuscitation and urgent procedure), 106 (18%) for cognitive impairment, 75 (13%) for behavioral affect, 18 (3%) for not speaking English, and 15 (3%) for surveys’ potential to interfere with standard of care. Among 192 potentially eligible patients, 116 agreed to complete the survey, while 76 declined. Reasons for declining participation were not systematically collected.

The median age of participants was 75 years (IQR 69–80), and most identified as women (53%), White (71%), and non-Hispanic (95%). Multimorbidity (ie, ≥2 chronic conditions) was highly prevalent (72%), with hypertension (75%) and high cholesterol (54%) as the most common conditions. Polypharmacy was also frequent: 37% of participants reported taking 5 to 9 medications daily, and 19% reported taking ≥10. Most individuals (85%) managed their medications independently, while smaller proportions relied on family (7%), informal caregivers (5%), or paid caregivers (3%).

Nearly half of the participants (47%) were college or postgraduate graduates, while 23% had completed high school, 20% attended some college, and 10% had less than a high school education. Reflecting this educational background, health literacy was generally high: 51% reported being “extremely” health literate and 26% “quite a bit.” Only a small proportion reported low literacy, with 6% indicating “not at all” and 1.7% noting they do not complete health care forms.

### 3.2 Attitudes Toward Deprescribing

A strong majority of ED patients expressed a willingness to deprescribe when consulted by their prescriber (91%; 95% CI: 84-95; Fig). Although slightly lower, a strong majority of patients were also willing to deprescribe when the recommendation came from ED staff (79%: 95% CI: 71-86). Despite this overall willingness, most ED patients reported satisfaction with their current medications (87%; 95% CI: 76-92). Patient demographics are further reported in Table 1.

**Figure:**
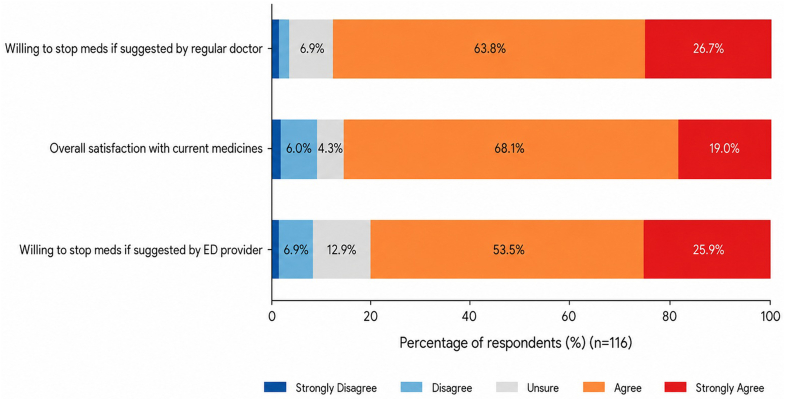
Older adults’ attitudes toward their medications and deprescribing in the emergency department.

**Table 1:** Participant demographics.

Characteristic	Frequency (n)	Percentage (%)
Age group, y		
65-74	56	48.3
75-84	47	40.5
≥85	13	11.2
Gender identity		
Woman	62	53.5
Man	44	37.9
Nonbinary	1	0.9
Prefer not to answer	9	7.8
Ethnicity		
Hispanic	4	3.5
Non-Hispanic	110	94.8
Prefer not to answer	2	1.7
Race		
White	82	70.7
Black	32	27.6
Asian	1	0.9
Prefer not to answer	1	0.9
Education level		
Some high school (or less)	11	9.5
Completed high school (12th grade)	27	23.3
< 4 y of college	23	19.8
College graduate or postgraduate	55	47.4
Health literacy		
Not at all	7	6.0
A little bit	3	2.6
Somewhat	15	12.9
Quite a bit	30	25.9
Extremely	59	50.9
N/A - I do not fill out forms	2	1.7
Multimorbidity		
Yes	83	71.6
No	33	28.5
Regular medications taken daily		
0	7	6.0
1–4	44	37.9
5–9	43	37.1
≥10	22	18.1
Medication Management		
Self-manage	99	85.3
Self-manage with family assistance	8	6.9
Managed by family/friend	6	5.2
Managed by a paid caregiver	3	2.6
Difficulty paying for medications		
Not at all	94	81.0
Somewhat	17	14.7
Extremely difficult	4	3.5
Unsure	1	0.9
Residence		
At home by yourself	40	34.5
At home with family/friends	70	60.3
Retirement community/village	5	4.3
Aged care facility	1	0.9

In our exploratory analyses on willingness to deprescribe by patient characteristics, demographic factors such as age, race, and gender identity did not significantly impact these attitudes (Table 2). Statistically significant differences in willingness were associated with the number of medications (*P* = .03) and confidence in completing medical forms (*P* = .04). Greater willingness to deprescribe was observed among patients taking more medications and those with lower confidence in completing medical forms.

**Table 2:** Participants’ willingness to deprescribe by demographic and clinical characteristics.

Characteristic	Strongly Disagree	Disagree	Unsure	Agree	Strongly Agree	*P* value^a^
Overall	1 (0.9)	2 (1.7)	8 (6.9)	74 (63.8)	31 (26.7)	
Age group, y						
65-74	1 (1.8)	0 (0.0)	6 (10.7)	34 (60.7)	15 (26.8)	0.40
75-84	0 (0.0)	2 (4.3)	2 (4.3)	29 (61.7)	14 (29.8)	
≥85	0 (0.0)	0 (0.0)	0 (0.0)	11 (84.6)	2 (15.4)	
Gender identity						
Woman	0 (0.0)	1 (1.6)	4 (6.5)	39 (62.9)	18 (29.0)	0.25
Man	1 (2.3)	0 (0.0)	3 (6.8)	32 (72.7)	8 (18.2)	
Nonbinary	0 (0.0)	0 (0.0)	0 (0.0)	1 (100.0)	0 (0.0)	
Prefer not to answer	0 (0.0)	1 (11.1)	1 (11.1)	2 (22.2)	5 (55.6)	
Race						
White	0 (0.0)	2 (2.4)	6 (7.3)	50 (61.0)	24 (29.3)	0.77
Black	1 (3.1)	0 (0.0)	2 (6.3)	23 (71.9)	6 (18.8)	
Asian	0 (0.0)	0 (0.0)	0 (0.0)	1 (100.0)	0 (0.0)	
Prefer not to answer	0 (0.0)	0 (0.0)	0 (0.0)	0 (0.0)	1 (100.0)	
Education						
Some High School	0 (0.0)	0 (0.0)	0 (0.0)	8 (72.7)	3 (27.3)	0.17
High School Grad	0 (0.0)	1 (3.7)	5 (18.5)	18 (66.7)	3 (11.1)	
< 4 Years College	1 (4.4)	0 (0.0)	1 (4.4)	15 (65.2)	6 (26.1)	
≥College Grad	0 (0.0)	1 (1.8)	2 (3.6)	33 (60.0)	19 (34.6)	
Confidence filling out medical forms						
Not at all confident	0 (0.0)	0 (0.0)	0 (0.0)	6 (85.7)	1 (14.3)	0.04
A little bit	0 (0.0)	1 (33.3)	0 (0.0)	1 (33.3)	1 (33.3)	
Somewhat	0 (0.0)	1 (6.7)	3 (20.0)	8 (53.3)	3 (20.0)	
Quite a bit	0 (0.0)	0 (0.0)	0 (0.0)	23 (76.7)	7 (23.3)	
Extremely	1 (1.7)	0 (0.0)	5 (8.5)	35 (59.3)	18 (30.5)	
Medication Count						
0	1 (14.3)	0 (0.0)	1 (14.3)	1 (14.3)	4 (57.1)	0.03
1-4	0 (0.0)	1 (2.3)	3 (6.8)	28 (63.6)	12 (27.3)	
5-9	0 (0.0)	1 (2.3)	3 (7.0)	29 (67.4)	10 (23.3)	
≥10	0 (0.0)	0 (0.0)	1 (4.6)	16 (72.7)	5 (22.7)	
Multimorbidity						
No	1 (3.0)	1 (3.0)	1 (3.0)	21 (63.6)	9 (27.3)	0.41
Yes	0 (0.0)	1 (1.2)	7 (8.4)	53 (63.9)	22 (26.5)	

a *P* value calculated using bivariate chi-squared tests.

### 3.3 rPATD Subdomains

Participant responses reflected a range of attitudes toward deprescribing across the four rPATD domains: *Burden*, *Appropriateness*, *Concerns about stopping*, and *Involvement*. Cumulative responses for each rPATD subdomain item are reported in Table 3.

**Table 3:** Likert-scale scores for Revised Patients’ Attitudes Towards Deprescribing (rPATD) subdomain items. All items were rated on a 5-point Likert scale (1 = strongly disagree to 5 = strongly agree).

rPATD subdomain	rPATD questionnaire item	Strongly disagree n (%)	Disagree n (%)	Neutral n (%)	Agree n (%)	Strongly agree n (%)
Burden	I spend a lot of money on my medicines.	23 (19.8)	54 (46.6)	6 (5.2)	25 (21.6)	8 (6.9)
	Taking my medicines every day is very inconvenient.	25 (21.6)	69 (59.5)	5 (4.3)	12 (10.3)	5 (4.3)
	I feel that I am taking a large number of medicines.	21 (18.1)	51 (44.0)	2 (1.7)	35 (30.2)	7 (6.0)
	I feel that my medicines are a burden to me.	24 (20.7)	70 (60.3)	6 (5.2)	13 (11.2)	3 (2.6)
	Sometimes I think I take too many medicines.	27 (23.3)	45 (38.8)	6 (5.2)	32 (27.6)	6 (5.2)
Appropriateness	I feel that I may be taking one or more medicines that I no longer need.	25 (21.6)	58 (50.0)	17 (14.7)	14 (12.1)	2 (1.7)
	I would like to try stopping one of my medicines to see how I feel without it.	26 (22.4)	50 (43.1)	8 (6.9)	25 (21.6)	7 (6.0)
	I would like my doctor to reduce the dose of one or more of my medicines.	24 (20.7)	60 (51.7)	13 (11.2)	15 (12.9)	4 (3.5)
	I think one or more of my medicines may not be working.	24 (20.7)	60 (51.7)	11 (9.5)	18 (15.5)	3 (2.6)
	I believe one or more of my medicines may be currently giving me side effects.	18 (15.5)	57 (49.1)	8 (6.9)	23 (19.8)	10 (8.6)
Concerns about stopping	I would be reluctant to stop a medicine that I had been taking for a long time.	9 (7.8)	34 (29.3)	12 (10.3)	51 (44.0)	10 (8.6)
	If one of my medicines was stopped, I would be worried about missing out on future benefits.	9 (7.8)	58 (50.0)	18 (15.5)	27 (23.3)	4 (3.5)
	I get stressed whenever changes are made to my medicines.	15 (12.9)	72 (62.1)	3 (2.6)	22 (19.0)	4 (3.5)
	If my doctor recommended stopping a medicine, I would feel that he/she was giving up on me.	40 (34.5)	69 (59.5)	1 (0.9)	6 (5.2)	0 (0.0)
	I have had a bad experience when stopping a medicine before.	19 (16.4)	74 (63.8)	7 (6.0)	12 (10.3)	4 (3.5)
Involvement	I have a good understanding of the reasons I was prescribed each of my medicines.	4 (3.5)	3 (2.6)	1 (0.9)	71 (61.2)	37 (31.9)
	I know exactly what medicines I am currently taking, and/or I keep an up-to-date list of my medicines.	3 (2.6)	3 (2.6)	2 (1.7)	70 (60.3)	38 (32.8)
	I like to know as much as possible about my medicines.	1 (0.9)	7 (6.0)	2 (1.7)	60 (51.7)	46 (39.7)
	I like to be involved in making decisions about my medicines with my doctors.	2 (1.7)	9 (7.8)	4 (3.5)	57 (49.1)	44 (37.9)
	I always ask my doctor, pharmacist or other health care professional if there is something I don't understand about my medicines.	2 (1.7)	9 (7.8)	3 (2.6)	58 (50.0)	44 (37.9)

#### 3.3.1 Burden factor

Overall, participants reported a low sense of burden from their medications. Most either disagreed or strongly disagreed with burden-related statements—66.4% disagreed that they spend a lot of money on medications, 81% did not find taking their medications daily to be inconvenient, and 64.7% disagreed that their medications feel burdensome. Only 32.8% of participants agreed or strongly agreed with the statement, “Sometimes I think I take too many medicines.”

#### 3.3.2 Appropriateness Factor

Attitudes toward the appropriateness of current medications were mixed. Although over half disagreed with the idea that they may be taking medications they no longer need, 14.7% were unsure. Nearly half (49.4%) were not interested in stopping a medicine to see how they felt without it. However, 28.5% acknowledged that ≥1 of their medications might be causing side effects.

#### 3.3.3 Concerns about stopping factor

Participants showed moderate concern about stopping a long-term medication. Just over half (52.6%; 95% CI: 43-62) said they would be reluctant to stop a medication they had been taking for a long time. Still, most participants (94%) rejected the idea that deprescribing meant their provider was “giving up” on them.

#### 3.3.4 Involvement factor

Participants reported high levels of engagement and awareness regarding their medications. A majority (93%) said they understood why each medication had been prescribed and knew what they were currently taking. In addition, 87% expressed a desire to be actively involved in decision making about their medications alongside their health care providers.

## 4 Limitations

This study is subject to several limitations. First, the sample was drawn from a single ED setting, which may limit the generalizability of findings to other settings or broader patient populations. Additionally, recruitment occurred only between 9:00 AM and 9:00 PM, Monday through Friday, based on the availability of study personnel. This recruitment strategy reflects a trade-off between external validity and the ability to engage patients during an active ED visit, allowing participants to reflect on deprescribing in the context of an acute care episode.

Second, the study population had disproportionately high education and health literacy compared with the broader population. This likely contributed to the high levels of engagement and medication awareness observed in the results. It also suggests that these patients may be more receptive to scientific reasoning and evidence-based guidance, potentially increasing their willingness to deprescribe. Consequently, these findings may overestimate willingness to deprescribe and limit applicability to patients with lower health literacy, who may require additional support and counseling—a notable challenge in the fast-paced ED.

Third, the exclusion of non-English-speaking patients likely reduced participation among certain minority groups and may have introduced selection bias. As a result, the study population may not reflect the full demographic of the cultural diversity of ED patients. The high rate of refusal of eligible patients may also have influenced study demographics; however, reasons for non-participation were not collected, which limits interpretation of potential selection bias.

Finally, all data were self-reported and therefore susceptible to social desirability or recall biases. Although rPATD offers valuable insight into participants’ attitudes toward deprescribing, it does not capture actual behaviors or outcomes. Stated willingness to deprescribe may not translate into real-world action when patients are presented with the opportunity to stop a medication. It is possible that fewer individuals would agree to deprescribe in practice than survey responses suggest.

Furthermore, the most common response across survey items was “agree”, raising the question of whether this reflects a meaningful endorsement or a more general acquiescence to survey participation within the medical environment, where patients may perceive research engagement as linked to their care. However, as noted above, patients were informed prior to survey administration that participation was voluntary and would not affect their care.

Additionally, the survey utilized in this study was an ED-augmented version of the psychometrically validated rPATD instrument. It included an additional exploratory question at the end of the survey and was administered in a clinical setting not previously validated for this instrument, potentially limiting validity. However, the validated rPATD domains were preserved without modification and analyzed independently from the exploratory item to maintain interpretability of the core constructs. Although this adaptation allows for novel application of the rPATD in the ED, it introduces the possibility that the clinical environment or added item may have influenced participant responses.

## 5 Discussion

This study provides valuable insight into the attitudes of older adults toward deprescribing in the ED setting. There was a strong overall willingness among ED patients to consider deprescribing, particularly when the recommendation comes from a trusted primary prescriber. Over 90% of participants reported being open to stopping one or more regular medications if advised by their primary prescriber, whether a primary care clinician or specialist; and notably, nearly 80% expressed similar openness when the suggestion came from ED staff. Although slightly fewer participants are receptive to deprescribing with ED staff, this still represents a majority. This suggests that the ED may be a viable setting to initiate deprescribing conversations, particularly when coordinated with outpatient follow-up. These findings are consistent with prior literature indicating that older adults are amenable to deprescribing across a range of populations, including those studied using the rPATD.^18^^,^^19^ To our knowledge, this is the first study to evaluate willingness to deprescribe among older adults in the ED setting. Furthermore, it is the first to demonstrate high willingness when the recommendation originates from an emergency medicine provider. This is reassuring, as prior efforts in ED-based medication review and deprescribing, though scarce, have shown feasibility.^20^ Our findings further suggest that such efforts are not only achievable but also desired by patients.

Across the rPATD domains, participants generally reported a low perceived medication burden. Most did not view their daily regimens as inconvenient or costly and did not consider themselves overmedicated. These findings suggest that patients may have normalized complex medication regimens as an expected part of aging or chronic disease management. This normalization could pose a future barrier to deprescribing conversations.

Although medications were not widely perceived as burdensome, nearly one-third of respondents expressed concerns about side effects. This suggests that even when patients accept their medication regimens, they may still worry about cumulative adverse effects. Side effect concerns are especially relevant to the ED, where older adults often present with acute complaints that may be medication-related, such as dizziness, falls, or altered mental status–conditions commonly linked to polypharmacy.^21^, ^22^, ^23^

Concerns about stopping long-standing medications were also evident, with over half of respondents expressing reluctance to discontinue drugs they had been taking for a long time. This reluctance likely reflects concern about the destabilization of chronic conditions, including the perceived loss of therapeutic benefit following medication discontinuation.^24^ However, such beliefs assume that a medication's anticipated benefits and harms remain static over time, whereas medication appropriateness is dynamic and may evolve with factors such as drug–drug interactions or declining creatinine clearance. Shared decision-making frameworks that reframe medication benefits and harms over time have been shown to increase deprescribing success.^25^^,^^26^ In the ED, time constraints and the need to address psychological barriers may limit opportunities for such nuanced discussions.

Despite overall willingness to deprescribe, most participants expressed overall satisfaction with their current medication regimens. This apparent contradiction suggests that satisfaction and willingness to deprescribe reflect two distinct constructs rather than mutually exclusive attitudes. Patients may be content with their current treatment yet remain receptive to change when guided by a trusted clinician.^27^ Satisfaction likely reflects contentment with the current treatment plan in the absence of new information, whereas willingness to deprescribe reflects responsiveness to clinician-led reassessment of medication appropriateness. This interpretation is supported by the strong emphasis participants placed on involvement in medication decision making, with most reporting a desire to play an active role in these decisions. Thus, satisfaction does not necessarily indicate resistance to change but rather comfort with current care in the absence of new clinical input. Prior studies have similarly shown that shared decision making and clear communication are critical components of successful deprescribing interventions rather than dissatisfaction with medications alone.^28^ This suggests that ED-based deprescribing interventions are unlikely to be limited by patient satisfaction or reluctance but rather depend on the presence shared decision-making tools and coordinated follow-up to support implementation.

Prior literature suggests that attitudes toward deprescribing may vary across patient subgroups, although findings are not always consistent.^19^ Although the present study was not powered to fully assess such variation, patients with higher levels of polypharmacy in our cohort reported greater openness to deprescribing, echoing previous associations between high medication burden and increased receptivity.^29^ This relationship may also underlie our observed association between decreased confidence in filling out medical forms and willingness to deprescribe; notably, lower educational attainment has been associated with a higher likelihood of polypharmacy, even after controlling for factors, such as age.^30^^,^^31^

Nonetheless, our findings suggest that patients remain willing to engage in deprescribing conversations with ED staff. As such, ED clinicians may appropriately raise concerns about medication appropriateness and initiate deprescribing discussions while coordinating with outpatient providers to support more comprehensive follow-up.

In summary, older adults receiving care in the ED demonstrate a high willingness to consider deprescribing, including when recommendations are initiated by emergency medicine providers. Although patients may express concerns about medication discontinuation and perceive limited burden from long-standing regimens, these findings suggest that the ED represents a meaningful opportunity to identify potentially inappropriate medications and initiate patient-centered deprescribing conversations. Future work should focus on developing scalable, ED-appropriate interventions that support shared decision making and ensure continuity with outpatient care to translate expressed willingness into safe and effective deprescribing practice.

## Author Contributions

EL, SB, PG, JN, JBW, MLM, and MFC developed the study concept and design. EL, GB, KD, AD, and GA were responsible for data acquisition and management. All authors contributed to data valdiation. EL and MFC drafted the manuscript and all authors contributed substantially to its revision. EL and MFC take responsibility for the manuscript as a whole.

## Funding and Support

By *JACEP Open* policy, all authors are required to disclose any and all commercial, financial, and other relationships in any way related to the subject of this article as per ICMJE conflict of interest guidelines (see www.icmje.org). Emma Lopes was supported by the 10.13039/100000062National Institute of Diabetes and Digestive and Kidney Diseases (T35DK007386). Martin F. Casey receives support from the 10.13039/100000049National Institute on Aging (R03AG089044). The content is solely the responsibility of the authors and does not necessarily represent the official views of the funding agency.

## Conflicts of Interest

All authors have affirmed they have no conflicts of interest to declare.
