# Supplementary material for: Attitudes Toward Deprescribing Among Older Adults Seeking Care in the Emergency Department
Source: J Am Coll Emerg Physicians Open. 2026 Jul 28;7(5):100467. doi: 10.1016/j.acepjo.2026.100467 (PMC13449356; doi:10.1016/j.acepjo.2026.100467)
Supplement: Supplementary file 1 [file mmc1.docx]

**Supplemental Figure S1.** Revised Patients’ Attitudes Towards Deprescribing (rPATD) prompts.

| Instructions  This questionnaire does not have right or wrong answers. Please mark the box with an X to indicate the extent to which you agree with each of the following statements. If there are any questions that you cannot answer, or believe that it does not apply to you, please skip them and move to the next question.  For questions that ask about your doctor, please think of the doctor who prescribes the most (if not all) of your medicines. | |
| --- | --- |
| P1 | I spend a lot of money on my medicines |
| P2 | Taking my medicines every day is very inconvenient. |
| P3 | I feel that I am taking a large number of medicines. |
| P4 | I feel that my medicines are a burden to me. |
| P5 | Sometimes I think I take too many medicines. |
| P6 | I feel that I may be taking one or more medicines that I no longer need. |
| P7 | I would like to try stopping one of my medicines to see how I feel without it. |
| P8 | I would like my doctor to reduce the dose of one or more of my medicines. |
| P9 | I think one or more of my medicines may not be working. |
| P10 | I believe one or more of my medicines may be currently giving me side effects. |
| P11 | I would be reluctant to stop a medicine that I had been taking for a long time. |
| P12 | If one of my medicines was stopped, I would be worried about missing out on future benefits. |
| P13 | I get stressed whenever changes are made to my medicines. |
| P14 | If my doctor recommended stopping a medicine, I would feel that he/she was giving up on me. |
| P15 | I have had a bad experience when stopping a medicine before. |
| P16 | I have a good understanding of the reasons I was prescribed each of my medicines. |
| P17 | I know exactly what medicines I am currently taking, and/or I keep an up-to-date list of my medicines. |
| P18 | I like to know as much as possible about my medicines. |
| P19 | I like to be involved in making decisions about my medicines with my doctors. |
| P20 | I always ask my doctor, pharmacist or other healthcare professional if there is something I don’t understand about my medicines. |
| P21 | If my doctor said it was possible I would be willing to stop one or more of my regular medicines. |
| P22 | Overall, I am satisfied with my current medicines. |
| P23* | If my emergency provider said one or more of my medicines were causing problems, I would be willing to stop them. |

** P23 is the EM-specific exploratory question and is not a psychometrically validated question.*
